# Children's myopia: prevention and the role of school programmes

**Published:** 2017-08-07

**Authors:** Catherine L. Jan, Clare Szalay Timbo, Nathan Congdon

**Affiliations:** 1 Centre for Brain and Cognitive Sciences, Peking University; Beijing Tongren Hospital, Beijing Capitol University, Beijing, China.; 2 Orbis International, New York, USA.; 3 Orbis International, New York, USA; Translational Research for Equitable Eyecare, Centre for Public Health, Queen's University Belfast, Belfast, Northern Ireland; Zhongshan Ophthalmic Centre, Sun Yat-sen University, Guangzhou, China.

**Exciting solutions are being developed to combat the huge increase in childhood myopia that had become the leading cause of visual impairment, particularly in East Asia. In these settings school-based vision care programmes can make a real difference.**

**Figure:**
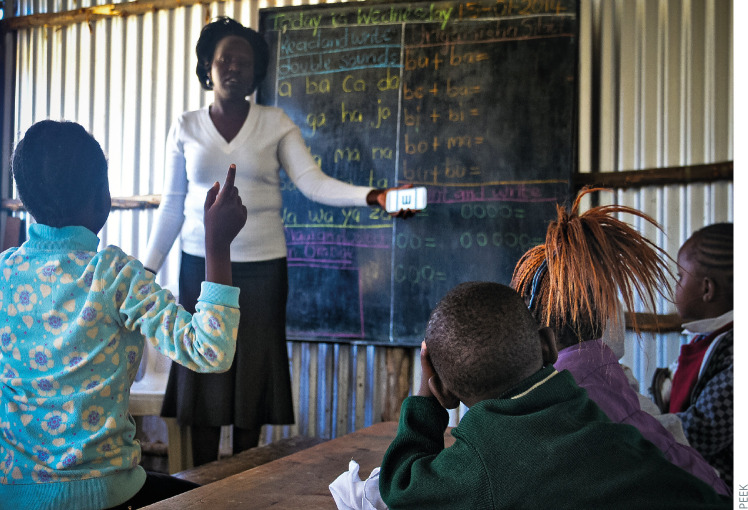
Teacher administering a vision test in a rural African school. KENYA

There are 12.8 million children worldwide who are visually impaired due to uncorrected refractive error (URE)^1^, the leading cause of visual impairment among children wherever the problem has been studied.^2^ Half of these children live in China^1^, where the total number with URE may reach 100 million by 2020.^3^ The prevalence of myopia, the most common refractive error, is growing rapidly in children around the world, reaching 80–90% among East Asian secondary school students.^4^

## Why we care

Spectacles are crucial to achieving the United Nation's Sustainable Development Goals on access to essential health care services and equitable, high-quality education.^5^ They provide an inexpensive, safe and effective means of addressing URE. Giving a child spectacles significantly improves educational outcomes^6^, unlocking a lifetime of opportunities.

**Figure:**
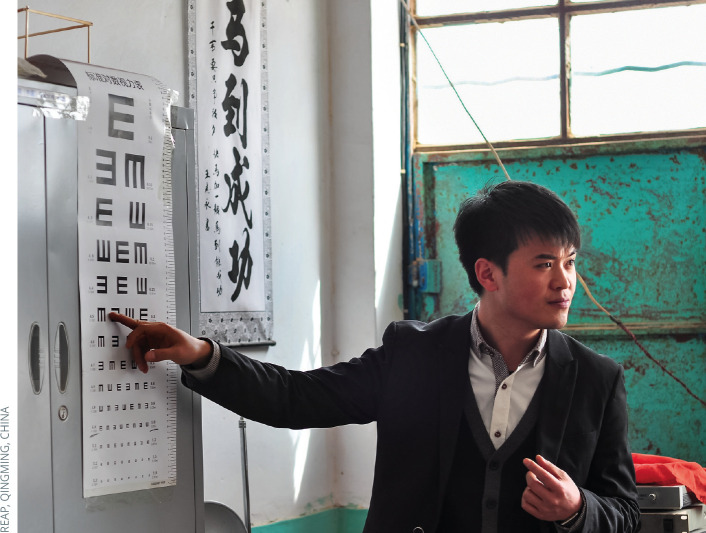
Vision testing by a teacher in a rural classroom. CHINA

## Challenges

Unfortunately, in areas of limited resources, as few as 15–25% of children who need spectacles actually have them.^6^,^7^ Reasons for this lost opportunity include the cost of spectacles (provision of free spectacles have been shown to double rates of use^6^); fear that spectacles harm children's vision, even though this has been proven to be incorrect;^8^ parents' lack of knowledge about their children's myopia; low rates of use when spectacles are given (which can be improved by various methods, including teacher incentives); the idea that wearing spectacles is unappealing or inconvenient; and the poor quality of available refractive services.^9^

## Solutions: reducing myopia in children

Exciting developments have recently occurred in the prevention of myopia through increased time spent outdoors^10^, multi- or dual-focal lenses, overnight hard contact lenses (Ortho-K), and use of very low concentration atropine eye drops. It appears that an additional 40 minutes per day spent outdoors can reduce new cases of myopia by a quarter^10^, and some studies suggest that more time outdoors might lead to even greater reductions, perhaps as much as a 50% decrease. In some countries, such as China, where myopia rates are very high, pressures for children to study more have made it difficult to increase the time spent outdoors. However, a full-scale, island-wide programme in Taiwan called ‘Daily 120’ has added two hours (120 minutes) of outdoor time to every school day for all children, and there are indications that myopia rates may be falling as a consequence. Increased outdoor time for children can also reduce the risk of diabetes and childhood obesity, two growing problems in children worldwide, and may also be helpful in combating vitamin D deficiency.

Regarding atropine, higher concentrations can cause blurred vision for reading and discomfort in bright lights due to dilatation of the pupils. However, recent studies in Singapore^11^ suggest that using very low concentrations (0.01%) offer several advantages: this dose has nearly as strong an effect in slowing myopia progression as higher concentrations, does not affect near vision, causes no problems with discomfort from bright lights, and – most importantly – does not appear to have a strong ‘rebound’ effect (an increase in myopia after cessation of the drops). Due to this latter reason, the overall effect of 0.01% atropine in reducing myopia may actually be greater than that of higher concentrations.

## Solutions: school screening programmes

Until these strategies are ready for wider use, schools provide an appealing location for carrying out traditional vision screening for children. As attendance rates continue to climb throughout the world, schools offer a convenient setting where the majority of children in a community may be found and regular follow-up can be provided, often with the assistance of teachers who are familiar with children's needs. Children attending school are more likely to develop myopia requiring the use of spectacles, and the educational benefits of spectacle wear are most likely to be realised if teachers help to support their use in school. Treatment for the full range of vision problems affecting children in a particular setting can be arranged by collaborating with nearby vision care facilities. School-based vision care programmes work best in settings with a larger burden of refractive error, a greater proportion of children attending school, a higher population density and better transport infrastructure. Below are two examples.

**The Rural Education Action Programme (REAP)'s Seeing for Learning social enterprise programme** is a successful collaboration between the private and public sectors. It provides vision screening services and spectacles to children living in rural areas in China. Teachers are trained to provide initial vision screening of students, and children who need additional care are referred to affiliated vision centres at nearby hospitals. After additional examinations and refraction by a medical professional at the vision centre, rural school children receive their first pair of spectacles free. The vision centre is able to access a new consumer market, children receive the services and spectacles they need, schools see improved test scores and the county government is credited with addressing a public health concern.

**Orbis' new REACH (Refractive Error Among Children) programme** is working with local partners to address the problem of URE among three million school-going children in fifteen districts across India. REACH Guidelines help standardise the screening process for all partners. The programme includes the use of LED pocket screeners, hand-held autorefractors and REACHSoft, a comprehensive software solution developed to capture data for planning, implementation, and monitoring of field activities in real time. The data generated are analysed and used to better understand local service delivery challenges, thereby aiding future programmes.

**Figure:**
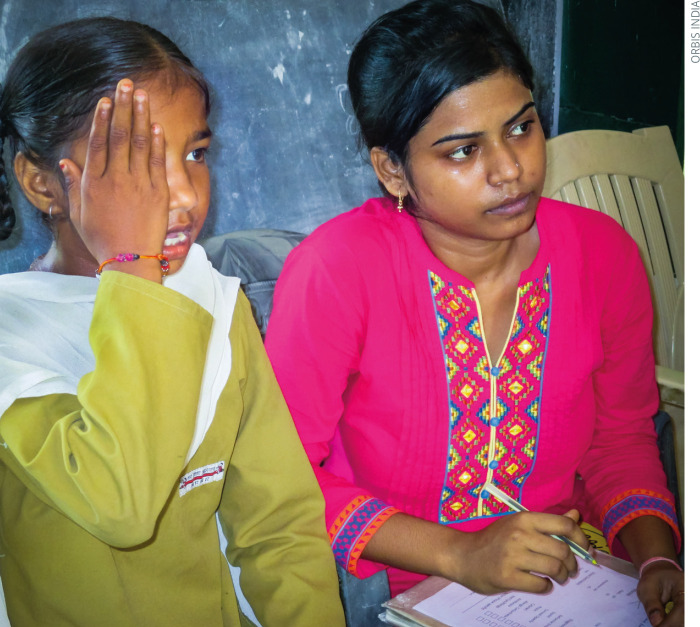
School vision screening. INDIA

These and many more school vision programmes around the world bring health care and education providers together to improve children's vision in the setting where it matters most: the schools in which they must see to learn.
